# Cis-9, Trans-11 CLA Alleviates Lipopolysaccharide-Induced Depression of Fatty Acid Synthesis by Inhibiting Oxidative Stress and Autophagy in Bovine Mammary Epithelial Cells

**DOI:** 10.3390/antiox11010055

**Published:** 2021-12-27

**Authors:** Nana Ma, Guozhen Wei, Hongzhu Zhang, Hongyu Dai, Animesh Chandra Roy, Xiaoli Shi, Guangjun Chang, Xiangzhen Shen

**Affiliations:** 1 Ministry of Education Joint International Research Laboratory of Animal Health and Food Safety, College of Veterinary Medicine, Nanjing Agricultural University, Nanjing 210095, China; 2017207035@njau.edu.cn (N.M.); 2018107097@njau.edu.cn (G.W.); 2018107096@njau.edu.cn (H.Z.); changguangjun@njau.edu.cn (G.C.); 2 College of Veterinary Medicine, Henan Agricultural University, Zhengzhou 450046, China; hongyud@henau.edu.cn; 3 Faculty of Veterinary, Animal and Biomedical Sciences, Sylhet Agricultural University, Sylhet 3100, Bangladesh; royanimeshvet98@yahoo.com; 4 College of Animal Science and Technology, Nanjing Agricultural University, Nanjing 210095, China; shixiaoli@njau.edu.cn

**Keywords:** LPS, cis-9, trans-11 CLA, oxidative stress, autophagy, milk fat metabolism, BMECs

## Abstract

Lipopolysaccharide (LPS) is the dominating endotoxin of Gram-negative bacteria, which can cause mastitis. Bovine mammary epithelial cells (BMECs), as major components of the mammary gland, usually suffer LPS challenge. Cis-9, trans-11 conjugated linoleic acid (CLA) has been reported to have anti-inflammatory characteristics, while its anti-oxidative ability to maintain cellular homeostasis in BMECs under LPS challenge is limited. Therefore, we studied whether cis-9, trans-11 CLA can restore the disturbance of cellular homeostasis indicated by the redox status and autophagy level caused by LPS and have an effect on cellular function- milk fat metabolism. For oxidative stress, LPS challenge promoted the formation of reactive oxygen species (ROS) and thiobarbituric acid reactive substances (TBARS) and decreased the concentration of glutathione. Anti-oxidative signaling regulated by transcription factor nuclear factor, erythroid 2 like 2 (Nrf2) was also depressed by LPS at the mRNA and protein level. However, cis-9, trans-11 CLA pretreatment downregulated the formation of ROS and TBARS and upregulated the expression of antioxidative enzymes. As a part of innate immunity, autophagy was also motivated by LPS challenge, while CLA decreased the autophagy level. LPS and H_2_O_2_ inhibited milk fat synthesis-related transcription factor sterol regulatory element binding protein (SREBP1), peroxisome proliferator activated receptor gamma (PPARG) and their downstream enzymes. Furthermore, 50 uM cis-9, trans-11 CLA promoted the mRNA and protein abundance of milk fat synthesis-related genes and lipid droplet formation in BMECs. In conclusion, LPS challenge disturbed the cellular homeostasis and depressed milk fat synthesis in BMECs; while cis-9, trans-11 CLA alleviated oxidative stress and decreased autophagy level, thus promoting milk fat synthesis, which offers a natural therapeutic strategy for mastitis.

## 1. Introduction

Mastitis is an inflammatory disease that occurs in the mammary gland, which reduces not only milk production but also milk quality [1]. There are various factors that induce mastitis, in particular, bacteria invasion [2]. Gram-negative bacteria, *Escherichia coli*, are identified as the leading bacteria triggering mastitis in dairy cows [3,4]. Lipopolysaccharide (LPS) is the main virulence factor on the surface of Gram-negative bacteria and is widely used to construct in vivo and in vitro mastitis models [5,6]. LPS can bind to its receptor, Toll-like receptor 4 (TLR4), and induce the key inflammatory transcript factor, nuclear factor κB (NF-κB), to translocate into the nucleus, leading to the production of a variety of pro-inflammatory cytokines, such as interleukin 1β (IL-1β), IL-6 and tumor necrosis factor α (TNF-α) [7].

The imbalance between the production of reactive oxygen species (ROS) and antioxidant system results in the accumulation of ROS, which is an important underlying cause of many health disorders, especially in the pre-parturition period [8]. The occurrence and development of oxidative stress contributes significantly to dysfunctional inflammatory response [9]. The usage of antioxidant supplementation, such as Se and Vitamin E, shortened the duration of mastitis, attenuated clinical symptoms and offered the more efficient clearance of inflammation, meaning that oxidative stress has a close relationship with the incidence of mastitis [10]. In addition to the application of antioxidants, the cellular antioxidant oxidase system also plays an important role in alleviating oxidative stress; Reduced glutathione (GSH) is a pivotal antioxidant that modulates cellular redox status [11]. There are three enzymes catalyzing the formation of GSH, GSH synthase (GSS), glutamate-cysteine ligase catalytic subunit (GCLC) and glutamate-cysteine ligase modifier subunit (GCLM). Nuclear factor, erythroid 2 like 2 (Nrf2), a transcription factor, is the core of the antioxidant system, as it can bind to antioxidant-response elements (AREs) in the promoters of antioxidant target genes, such as superoxide dismutase (SOD), glutathione biosynthetic enzymes (GCLC and GCLM), glutathione peroxidase (GPX), glutathione S-transferases (GST) and heme oxygenase-1 (HMOX1) et al. [12]. Autophagy is a highly conserved physiological process, which participates in subjecting cellular homeostasis to a series of challenges [13], such as hypoxia/reoxygenation [14] or hyperketonemia [15]. Autophagy can also limit the epithelial inflammatory response to kidney injury by suppressing NF-κB signaling [16]. Given the close relationship between oxidative stress/autophagy and inflammation, redox status and autophagy were considered as indicators of the cellular homeostasis of bovine mammary epithelial cells.

Conjugated linoleic acid (CLA) is an octadecadienoic acid characterized by two conjugated double bonds, mainly including two isomers, trans-10, cis-12 CLA and cis-9, trans-11 CLA. Many studies have shown that CLA exhibits many important bioactive functions, such as anticancer, inhibition of atherosclerosis, reduction in body fat [17] and modulation of immune response [18,19]. N Hanschke et al. explored the antioxidative ability of CLA (equal content of cis-9, trans-11 CLA and trans-10, cis-12 CLA); the concentration of thiobarbituric acid-reactive substances (TBARS) was reduced by CLA management, meaning that the dietary supplementation of CLA has an antioxidative ability in dairy cows during d1 to 182 postpartum [20]. CLA isomers also have the ability to increase the activity of anti-oxidative stress enzymes SOD, GPX and GST, and to decrease the concentrations of intracellular ROS and TBARS in BMECs treated with H_2_O_2_ [21]. Cis-9, trans-11 CLA also improved duodenal redox status by activating Nrf2 to mediate its defense against gliadin-induced enteropathy in mice [22] and had a cytoprotective ability in mouse dendritic cells under LPS or gliadin challenge through the Nrf2 signaling pathway [23].

An important function of mammary epithelial cells is the production of milk fat. Rumen LPS has a strong relationship with the depression of milk fat content and yield, which may include the increased concentration of plasma C-reactive protein (CRP) [24]. LPS also reduces triglyceride synthesis via transcript factor sterol regulatory element binding protein 1 (SREBP1) in bovine mammary epithelial cells [25]. The disturbance of cellular homeostasis may significantly affect the cellular function, such as fatty acid synthesis. Many studies have proved the contribution of trans-10, cis-12 CLA to milk fat depression [26,27,28]. Both CLA isomers showed the ability to inhibit lipogenesis through reducing the mRNA expression and activity of Acetyl-CoA carboxylase alpha (ACACA) and stearoyl-CoA desaturase (SCD) in the mammary glands of lactating mice, and the ability of trans-10, cis-12 CLA was more potent than cis-9, trans-11 CLA [29]. Cis-9, trans-11 CLA was reported to ameliorate steatosis induced by a high-fat diet partly through the Nrf2 pathway in Wistar rats [30]. However, research on the effect of cis-9, trans-11 CLA on fatty acid metabolism in the mammary glands of dairy cows is limited.

Therefore, de novo synthase (ACACA, fatty acid synthase (FASN) and Acyl-CoA synthetase short chain family member 2 (ACSS2)), long-chain fatty acid converting enzymes (Acyl-CoA synthetase long-chain family member 1 (ACSL1) and SCD), fatty acids transporters (CD36, solute carrier family 27 member 1 (FATP-1), fatty acid binding protein 3 (FABP3) and Lipoprotein lipase (LPL)), triglyceride synthase (glycerol-3-phosphate acyltransferase 3 (GPAT3), 1-acylglycerol-3-phosphate O-acyltransferase 6 (AGPAT6), diacylglycerol O-acyltransferase 1 (DGAT1) and DGAT2), lipid droplet-releasing enzyme (butyrophilin subfamily 1 member A1 (BTN1A1) and perilipin 1 (PLIN1)) and transcription factors SREBP1 and peroxisome proliferator activated receptor gamma (PPARG) were measured to explore the effects of LPS and cis-9, trans-11 CLA. Meanwhile, antioxidant transcription factor Nrf2, its negative regulated factor: Kelch-like ECH-associated protein 1 (KEAP1) and cullin 3 (CUL3); and Nrf2 downstream target genes malic enzyme 1 (ME1), HMOX1, NAD(P)H quinone dehydrogenase 1 (NQO1), catalase (CAT), thioredoxin (TXN), thioredoxin reductase 1 (TXNRD), glutathione S-transferase mu 1 (GSTM1), glutathione-disulfide reductase (GSR) and GSS are responsible for the cellular antioxidant ability. The autophagy level was indicated by symbolic protein expression microtubule-associated protein 1 light chain 3 beta (LC3), accumulation of sequestosome 1 (p62), exthe pression of autophagy regulatory proteins Beclin1 and autophagy-related 5 (ATG5) and lysosome quality. We hypothesize that LPS challenge induces the cellular homeostasis disturbance indicated by oxidative stress and an increased autophagy level and destroyed cellular function-depression of fatty acid synthesis, while cis-9, trans-11 CLA supplementation can reverse these effects in bovine mammary epithelial cells.

## 2. Materials and Methods

### 2.1. Reagents and Chemicals

LPS from *E. coli* 055: B5 was purchased from Sigma-Aldrich (Burlington, MA, USA). Cis-9, trans-11 CLA was purchased from NU-CHEK Prep Inc., (UC-60A-D12-X, Elyslan, MN, USA) with a purity > 90% and >100 mg.

### 2.2. Bovine Mammary Epithelial Cell Culture

Bovine mammary epithelial cells (BMECs) were purchased from Tongpai Biological Technology Co., Ltd. (Shanghai, China), which was established and characterized by Ke Zhao et al. [31]. BMECs were well cultured in our experiment and used as a perfect in vitro model in several papers [32,33,34]. BMECs were cultured in complete medium, which contains 90% Roswell Park Memorial Institute (RPMI) medium 1640 basic (1×), 10% fetal bovine serum, 100 U/mL penicillin, and 100 μg/mL streptomycin and placed in a 37 °C humidified cell incubator with 5% CO_2_.

### 2.3. Experimental Design

First, BMECs were treated with LPS for different time to explore its effect on oxidative stress and fatty acid synthesis according to the mRNA expression of Nrf2, NQO1, HMOX1, SOD1, CAT for oxidative stress, and SREBP1, PPARG, ACACA and SCD for fatty acid synthesis. Then, 50 μM and 100 μM cis-9, trans-11 CLA, according to our previous experiment [32], were chosen to explore their anti-oxidative stress ability and effect on fatty acid synthesis at different times through qPCR and oil red stain. There were six groups: the control (CON, *n* = 3) group, H_2_O_2_ (positive control, 50μM for 3 h, *n* = 3) group, DMSO (reagent control, 50 μM for 24 h, *n* = 3) group, CLA treatment (50 μM for 24 h, *n* = 3) group, LPS challenge (8 μg/mL for 12 h, *n* = 3) group, CLA pretreatment followed by LPS challenge (CLPS, *n* = 3) group in the measurement of ROS by flow cytometry. Four groups: CON, CLA, LPS and CLPS, were used to confirm the anti-oxidative stress ability and effect on the fatty acid metabolism of CLA caused by LPS through assay kits, qPCR, oil red stain, WB and immunofluorescence analysis. For the CLPS group, cells were pretreated with 50 μM CLA for 24 h followed by 8 μg/mL LPS treatment for 12 h. 

### 2.4. Cell Viability

The existence of toxic effects in the concentrations of LPS used in this experiment on BMECs was checked by cell viability. Approximately 5×10^3^ BMECs were seeded in each well of a 96-well plate and cultured with 100 μL complete medium overnight. Then, the complete mediums with 0, 10, 100, 1000, and 10,000 ng/mL LPS were used to treat BMECs for 48 h. After each well was washed with 1 × PBS twice, 10 μL CCK-8 reagent from a Cell Counting Kit-8 (CCK-8) (Solarbio Life Sciences, Beijing, China) was added into 100 μL complete medium of each well. BMECs were cultured for 3 h, and the OD value of each well was measured at 450 nm wavelength with a full wavelength microplate photometer (Thermo Fisher Scientific Inc., Waltham, MA, USA).

### 2.5. Determination of CAT, GSH, TBARS and SOD

The content of TBARS partially indicates the degree of lipid peroxidation in tissue [35] and need to be combined with antioxidant kinase CAT and SOD and pivotal antioxidant GSH to reflect the cellular redox state. Approximately 1 × 10^5^ BMECs were seeded into 6-cm plates. After four treatments (as CON, CLA, LPS and CLPS group in experiment design), the supernatant was discarded, and cells were washed with PBS twice. Cells were collected with scraper and resuspended with 500 μL cold PBS. The suspension was lysed 4 times with ultrasound in ice at 300 W, once every 3–5 s, with intervals of approximately 30 s. The homogenates were saved in −20 °C for measurement. The activity of CAT and SOD and the concentration of GSH and TBARS were detected by commercial kits (Cat. No. A007-1-1 CAT, A001-3 SOD, A006-2-1 GSH, A003-1 TBARS, Jiancheng Bioengineering institute, Nanjing, China,). More description about methods of these kits was offered in Supplementary Table S1. The samples were prepared according to the introductions of each kit and the final results, OD values, were assayed by a full wavelength microplate photometer (Thermo Fisher Scientific Inc., Waltham, MA, USA). The related protein concentrations of each sample were detected using a bicinchoninic acid (BCA) protein assay kit (Pierce, Rockford, IL, USA).

### 2.6. Flow Cytometry for Reactive Oxygen Species

The ROS inside cells was assayed by an ROS assay kit (E004, Jiancheng Bioengineering institute, Nanjing, China). BMECs were spread onto 12-well plate. When they grew 50–60% confluence, cells were treated according to the experimental design. As the positive control, the concentration of the working solution of H_2_O_2_ was 50μM, and its working time was 3 h. After treatment, 2, 7-dichlorofuorescin diacetate (DCFH-DA) was added into medium at a concentration of 10μM for 1 h. Cells were digested with 0.25% trypsin, centrifuged with 1000 g for 5–10 min, and collected in a 1.5 mL EP tube. After being washed with PBS twice, cells were suspended with 500 μL PBS. The fluorescence intensity of ROS was measured with a BD FACSVerse™ 273 Flow Cytometer (BD Biosciences, Franklin Lakes, NJ, USA) at 500 or 485 (500 ± 15 nm) of the optimal excitation wavelengths and 525 (530 ± 20 nm) of the optimal emission wavelengths, and the data were analyzed using FlowJo. The test was divided into 7 groups, namely, the non-DCFH-DA group (DCFH-DA-), only DCFH-DA group without any treatment (DCFH-DA + CON), the reagent control DMSO group (DCFH-DA + DMSO), the positive control H_2_O_2_ group (DCFH-DA + H_2_O_2_), 50 μM CLA treatment for 24 h group (DCFH-DA + CLA), 8 μg/mL LPS treatment for 12 h group (DCFH-DA + LPS) and 50 μM CLA pre-treatment for 24 h and then 8 μg/mL LPS treatment for 12 h group (DCFH-DA + CLPS).

### 2.7. Oil Red for Lipid Droplets

The lipid droplets stained by oil red showed the content of milk fat synthesis. BMECs were grown on circular coverslips in the wells of 12-well plates. After treatment, the coverslips were airdried. The coverslips were stained with an oil red O dye solution commercial kit (D027-1, Jiancheng Bioengineering institute, Nanjing, China). After staining, the coverslips were placed on slides and fixed with mounting medium contained in the kits. Lipid droplets were observed under light microscopy and the images were observed with a high-resolution digital camera and NIS Elements F 3.0 image acquisition 192 software (Nikon Corporation, Minato-ku, Tokyo, Japan). Lipid was dyed red, the cell nucleus was dyed dark blue, and the other section of the cell was dyed light blue.

### 2.8. Extraction of Total RNA and Quantitative Real-Time Polymerase Chain Reaction (RT-qPCR)

BMECs were seeded in 24-well plate and divided into different groups according to the experimental design. When treatments ended, RNA iso Plus™ reagent (Takara Co., Otsu, Japan) was used to extract the total RNA from cells according to the manufacturer’s protocol. The quality of total RNA was assessed by both 1% agarose gel electrophoresis and a NanoDrop ND-1000 Spectrophotometer (Thermo Scientific, Waltham, MA, USA). Reverse transcription of total RNA to complementary DNA (cDNA) was conducted following the protocol of HiScript III RT SuperMix (R323-01, Vazyme biotech co., Itd., Nanjing, China). All primers used in this experiment are listed in Supplementary Table S2. The complementary DNA of every sample was diluted by 4-fold, and the RT-qPCR was performed using a 7300 real-time PCR system (Applied Biosystems, Foster City, CA, USA) using ChamQ Universal SYBR qPCR Master Mix (Q711, Vazyme Biotech Co., Itd., Nanjing, China). GAPDH was chose as the house keeping gene to normalize the data of each target genes. The relative expression of each target gene was analyzed using the 2^−ΔΔCt^ method as described before [36]. Results were presented as the fold change relative to the control.

### 2.9. Western Blotting Analysis

After treatment, cells were washed with PBS twice and the total protein of cells was extracted using RIPA Lysis buffer (Beyotime, Shanghai, China) premixed with 1 mM proteinase inhibitor PMSF. The protein concentration of cell lysis was detected using a BCA protein assay kit. Then, samples were diluted to the same concentration and denatured with 5× SDS loading buffer (BL502A, Biosharp life Sciences, Hefei, China). Protein was subjected to 10% SDS-PAGE by electrophoresis and transferred onto a polyvinylidene fluoride (PVDF) membrane. Membranes were incubated in 7% skimmed milk for 2 h at RT followed by incubated with primary antibodies at 4 °C overnight. After being washed with 1× Tris-buffered saline containing Tween 20 (TBST), membranes were transferred into horseradish peroxidase (HRP)-conjugated secondary antibodies. All information on the primary and secondary antibodies are listed in Supplementary Table S3. Bands on the membrane were visualized with an ECL Plus Kit (Vazyme biotech co., Itd., Nanjing, China), and the signals were captured by a ChemiDoc MP system (Bio-rad, Berkeley, CA, USA). The intensity of each band was analyzed with Image Lab software (Bio-rad, Berkeley, CA, USA). Glyceraldehyde-3-phosphate dehydrogenase (GAPDH) or β-actin was the reference protein to normalize the result of the target protein. The final result of each target protein was presented as relative abundance to GAPDH or β-actin. 

### 2.10. Immunofluorescence Analysis

BMECs were seeded onto the circular coverslips in 12-well plates. When cells grew 70–80% confluence, they were treated according to the experimental design. Cells were fixed with 500 μL/well 4% paraformaldehyde (Sigma-Aldrich, Burlington, MA, USA) for 20 min. The cell morphology on the coverslips was checked under a microscope. Cells were incubated with 0.3% Triton X-100 (500 μL/well, T9284, Solarbio Life Sciences, Beijing, China) for 15 min at RT to increase permeability. Blocking agent (1 × PBS/5% BSA/0.3% Triton X-100) was used to block cells at RT for 1 h. The primary antibodies, which are listed in Supplementary Table S2, were diluted in antibody buffer (1 × PBS/1% BSA/0.3% Triton X-100) and were used to incubated cells at 4 °C overnight. Then, cells were incubated with the FITC-labeled secondary antibodies, which are listed in Supplementary Table S2, and counterstained with DAPI (D8417, Sigma Aldrich, Burlington, MA, USA) to stain the nucleus for 5 min. The coverslips were fixed on glass slides by Antifade Mounting Medium (P0128, Beyotime, Shanghai, China). The fluorescent images of each target protein and nucleus inside the cells were visualized by an LSM 710 confocal laser microscope system (Zeiss, Oberkochen, Germany).

### 2.11. Statistical Analysis

All experiments were repeated three times independently with triplicates in each treatment. All data in this study were analyzed by one-way ANOVA with Duncan’s post-hoc test by IBM SPSS 20.0 Statistics for Windows (IBM Inc., New York, NY, USA). Before difference comparison, the normality distribution of the variables was checked by the Shapiro-Wilk test of SPSS. Results were presented as the mean and standard error of the mean (mean ± SEM). Significance was set at *p* ≤ 0.05.

## 3. Results

### 3.1. LPS Caused Oxidative Stress in BMECs

BMECs were stimulated with 8 μg/mL LPS for 2, 4, 6, 8, 12 and 24 h to check the influence of LPS on the mRNA expression of the antioxidant system. The mRNA levels of transcription factor Nrf2 and antioxidant kinase HMOX1 were significantly decreased from 4 h to 8 h, and increased to 24 h, which was not different from the control group. However, the mRNA expression of antioxidant kinases CAT and SOD1 was significantly higher between 4 h and 12 h than that in the control group. The mRNA abundance of NQO1 at only 24 h was significantly higher than that of the control group (Supplementary Figure S1a–e). The cell viability during treatment with 10–10,000 ng/mL LPS for 24 h was not influenced by LPS (Supplementary Figure S1f).

### 3.2. CLA Relieved Oxidative Stress Caused by LPS

**Figure 1:**
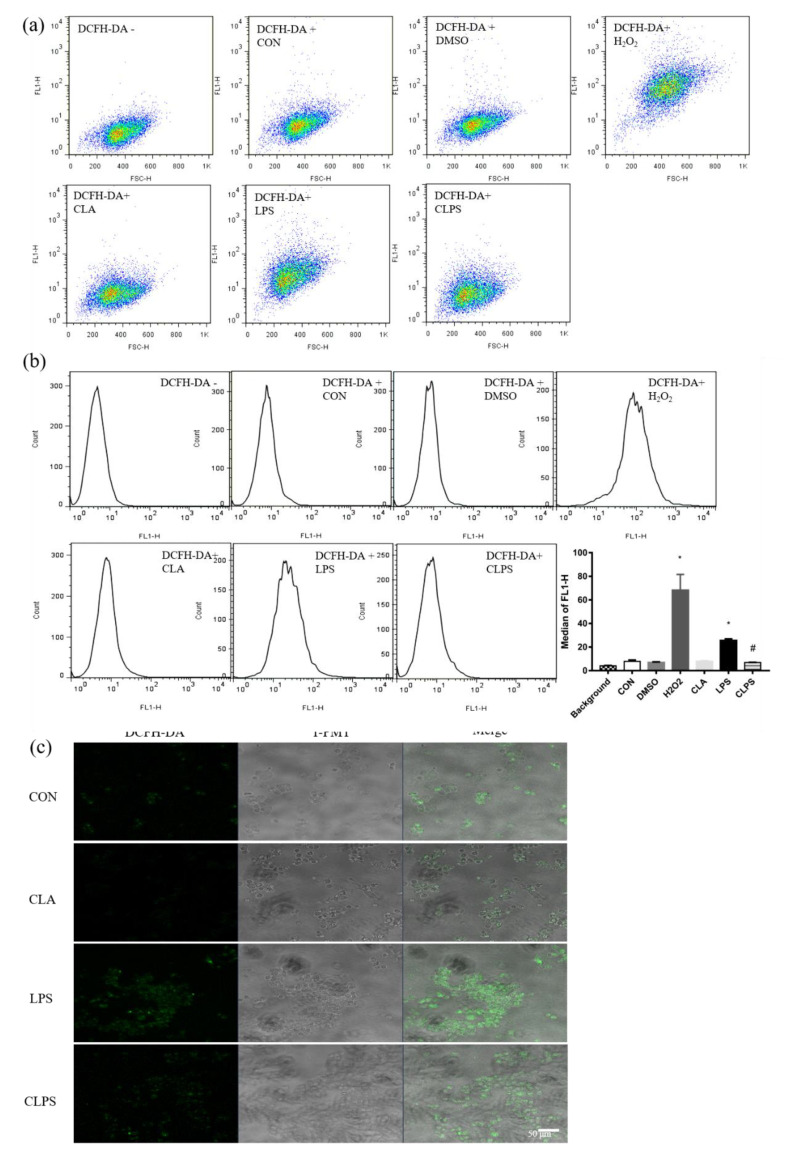
CLA pretreatment ameliorated the accumulation of reactive oxygen species (ROS) caused by LPS challenge in BMECs. (**a**) BMECs distribution diagram marked with green fluorescence probe DCFH-DA, which accurately represents the ROS concentration precisely. (**b**) The fluorescence intensity histogram, and the median of the fluorescence intensity was statistically analyzed. The data are presented as the means ± SEM. Experiments were repeated three times independently with triplicates in each treatment. * *p* ≤ 0.05, significantly different from CON; # *p* ≤ 0.05, significantly different from LPS. (**c**) ROS was marked with green fluorescence probe DCFH-DA and visualized by laser confocal microscopy. The T-PMT model was used to observe cell morphology and positioning.

Pretreatment with 50μM CLA from 24 h to 48 h can significantly upregulated the mRNA expression of Nrf2 in BMECs challenged by LPS. Additionally, the mRNA abundance of NQO1 was also increased by CLA pretreatment from 12 h to 24 h followed by LPS treatment in (Supplementary Figure S1g,h). Flow cytometry was applied to measure the ROS concentration in BMECs (Figure 1a,b), and LPS challenge significantly induced the production of ROS, while CLA reduced the concentration of ROS to the level of the control group. The green fluorescence of DCFH-DA is a specific probe for ROS. LPS stimulation significantly increased ROS accumulation in BMECs, while CLA pretreatment restored the content of ROS (Figure 1c).

**Figure 2:**
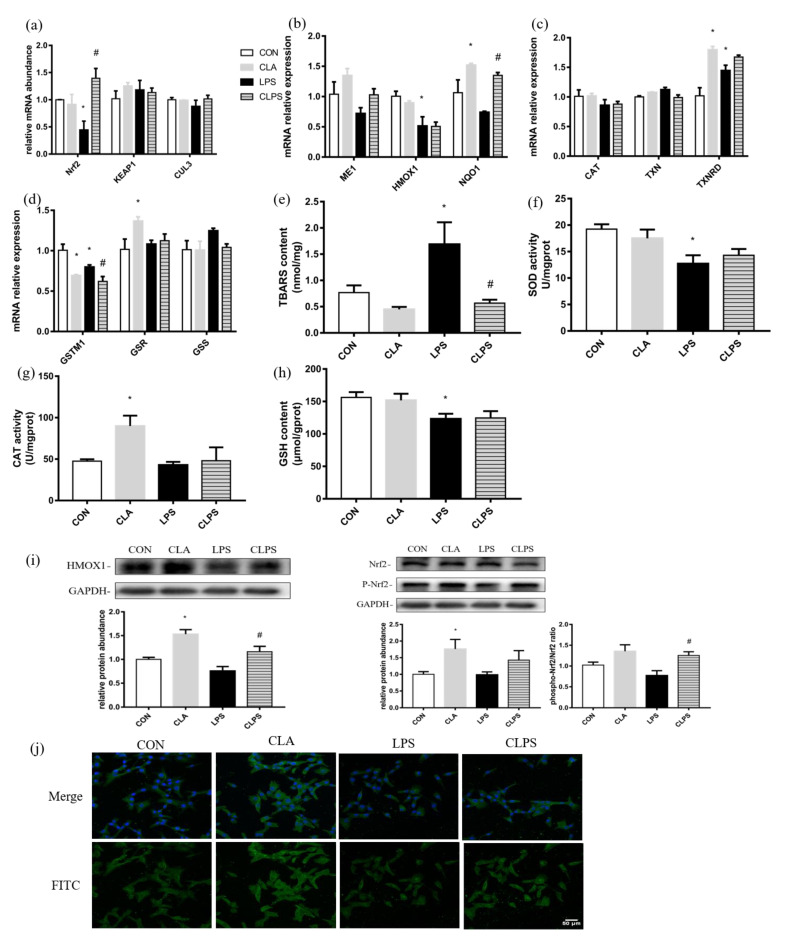
CLA pretreatment alleviated oxidative stress induced by LPS challenge in BMECs. (**a**–**d**) mRNA abundance of antioxidative genes (**e**–**h**) the activity of anti-oxidative enzymes by commercial kits (**i**) immunoblotting of HMOX1 and Nrf2 by Western blotting (**j**) Nrf2 visualized by FITC fluorescence (green), and nuclei visualized by DAPI (blue) through immunofluorescence analysis were measured to study the effect of CLA pretreatment and LPS challenge on the redox state of BMECs. The data are presented as the means ± SEM. Experiments were repeated three times independently with triplicates in each treatment. * *p* ≤ 0.05, significantly different from CON; # *p* ≤ 0.05, significantly different from LPS.

LPS stimulation could significantly reduce the mRNA expression of Nrf2, HMOX1 and GSTM1 in BMECs. Compared with the control group, CLA treatment alone could significantly increase the mRNA expression of NQO1, TXNRD and GSR. Compared with the LPS group, the mRNA expression of Nrf2 and NQO1 in the CLPS group was significantly increased (Figure 2a–d).

LPS stimulation significantly increased the TBARS content in cells, and CLA pretreatment restored the TBARS content to normal. LPS stimulation significantly reduced the content of antioxidant kinase SOD and the active substance GSH. CLA treatment alone could significantly increase the activity of the antioxidant kinase CAT (Figure 2e–h).

CLA treatment could significantly increase the Nrf2 activation state- phosphorylated Nrf2 and the protein expression of HMOX1. CLA pretreatment could significantly increase the ratio of phosphorylated Nrf2/total Nrf2 and the protein expression of HMOX1 which were reduced by LPS stimulation in BMECs (Figure 2i). CLA treatment increased the fluorescence intensity of Nrf2. LPS challenge reduced the fluorescence intensity of Nrf2, while CLA pretreatment under LPS could alleviate this phenomenon (Figure 2g).

### 3.3. CLA Pretreatment Decreased Autophagy Level Induced by LPS Challenge

**Figure 3:**
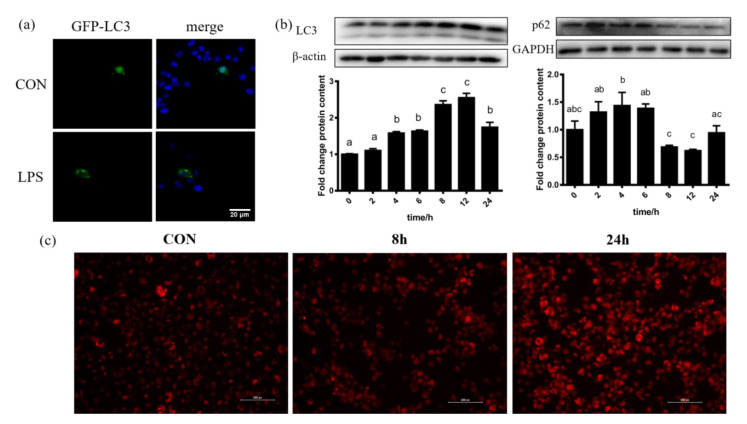
LPS challenge stimulated autophagy in BMECs. (**a**) BMECs transfected with GFP-LC3 plasmid expressed more green blots after challenged with LPS. (**b**) Immunoblotting of LC3B and p62 measured by Western blotting under treatment of LPS for 0, 2, 4, 6, 8, 12 and 24 h showed the occurrence of autophagy flow. (**c**) The fluorescence intensity of lysosomes in BMECs stained by red fluorescent probe Lyso-Tracker Red increased with the increase in LPS challenge time. The data are presented as the means ± SEM. Experiments were repeated three times independently with triplicates in each treatment. Different letters between groups represent significant differences with *p* ≤ 0.05.

BMECs that were transfected with GFP-LC3 expressed more LC3 under LPS challenge (Figure 3a). With the increase in the duration of LPS challenge, the protein abundance of LC3B increased, and the protein abundance corresponding to 8 h and 12 h was the highest. While the protein expression of p62 corresponding to 8 h and 12 h was the lowest (Figure 3b). The fluorescence intensity of lysosomes in BMECs stained by red fluorescent probe Lyso-Tracker Red was increased after LPS treatment (Figure 3c). LPS treatment increased the expression of LC3 and the formation of lysosome, and decreased the expression of p62, which reflected the enhancement of autophagy flow.

**Figure 4:**
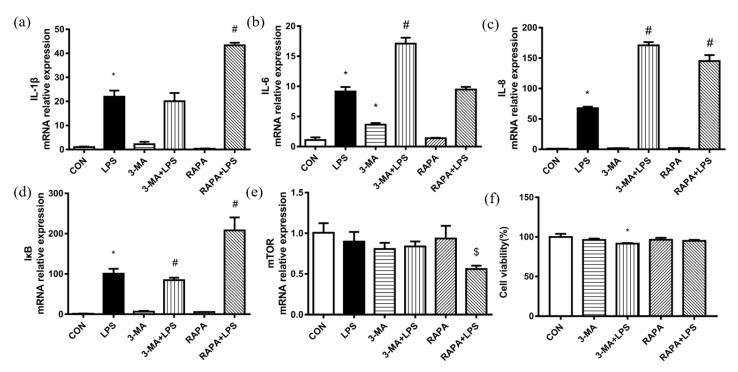
Effect of autophagy on inflammatory response of BMECs induced by LPS challenge. Effect of 3-MA (autophagy inhibitor) and rapamycin (autophagy inducer) on (**a**–**c**) mRNA expression of inflammatory cytokines (IL-1β, IL-6 and IL-8), (**d**) transcription factor (IκB), (**e**) mTOR and (**f**) cell viability. CON, control group. LPS, 8μg/mL LPS for 12 h. 3-MA, 5 mM 3-MA for 4 h. RAPA, 0.2μM rapamycin for 24 h. The data are presented as the means ± SEM. Experiments were repeated three times independently with triplicates in each treatment. * *p* ≤ 0.05, significantly different from CON; # *p* ≤ 0.05, significantly different from LPS.; $ *p* ≤ 0.05, significantly different from RAPA.

The mRNA expression of IL-1β, IL-6, IL-8 and IκB was upregulated by LPS challenge, and the depression of autophagy by 3-MA exacerbated the mRNA abundance of IL-6 and IL-8. Rapamycin (RAPA) treatment aggravated the mRNA abundance of IL-1β, IL-8 and IκB (Figure 4a–d). The mRNA abundance of the mammalian kinase target of rapamycin (mTOR) in the RAPA + LPS group was lower than that in the RAPA group (Figure 4e). Only 3-MA and LPS treatment simultaneously reduced cell viability more significantly than in the CON group, but the cell viability of the 3-MA + LPS group was more than 90% (Figure 4f). In conclusion, autophagy inhibitor 3-MA stimulated BMECs to express more inflammatory cytokines, meaning that the inhibition of autophagy in BMECs exacerbated the inflammatory response caused by LPS. While under LPS challenge, rapamycin downregulated the mRNA abundance of mTOR, however it promoted the mRNA abundance of some pro-inflammatory cytokines.

**Figure 5:**
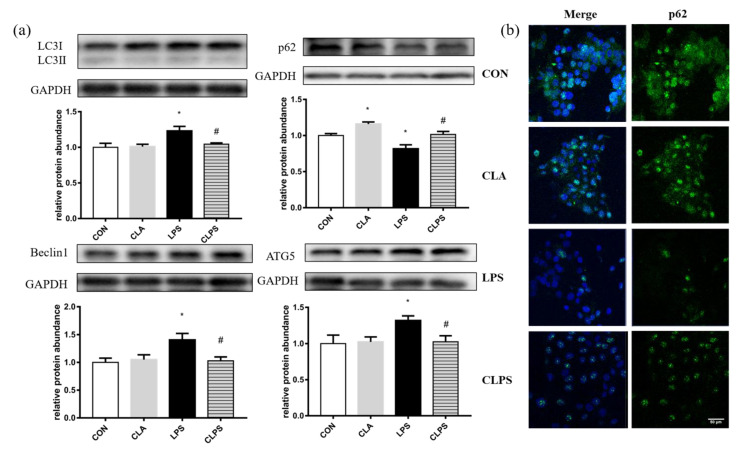
CLA pretreatment relieved the autophagy level stimulated by LPS challenge in BMECs. (**a**) Immunoblotting of autophagy related protein, LC3, p62, beclin 1 and ATG5 was measured by Western blotting. GAPDH was the reference protein to normalize the result of target proteins. The data are presented as means ± SEM. Experiments were repeated three times independently with triplicates in each treatment. * *p* ≤ 0.05, significantly different from CON; # *p* ≤ 0.05, significantly different from LPS. (**b**) p62 expression stained with FITC fluorescence (green) was visualized by immunofluorescence, and nuclei were stained with DAPI (blue).

The protein abundance of autophagy related proteins, including LC3B, Beclin 1 and ATG5, was upregulated by LPS challenged, while CLA pretreatment reversed this effect (Figure 5a). The protein abundance and immunofluorescence intensity of p62 (Figure 5a,b) was decreased by LPS treatment, while CLA pretreatment increased the protein abundance and immunofluorescence intensity of p62.

### 3.4. LPS and H_2_O_2_ Depressed Fatty Acid Synthesis

**Figure 6:**
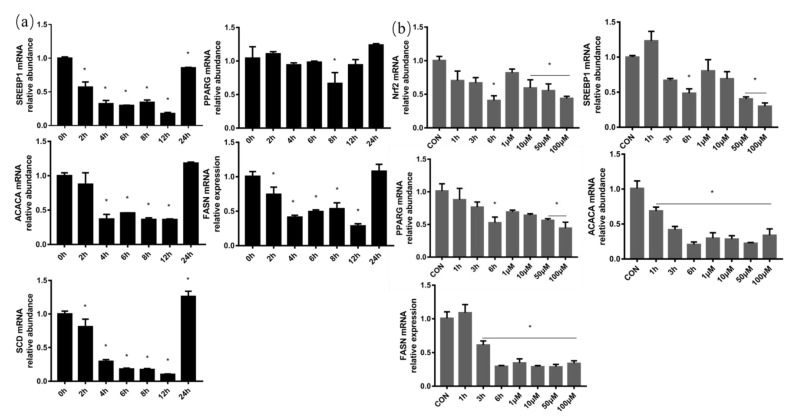
LPS and H_2_O_2_ treatment depressed mRNA abundance of milk fat synthesis-related genes in BMECs. (**a**) The mRNA abundance of milk fat synthesis-related transcription factor (SREBP1 and PPARG) and milk fat synthase (ACACA, FASN and SCD) in BMECs was measured under treatment with 8μg/mL LPS for 0, 2, 4, 6, 8, 12 and 24 h. (**b**) Oxidative stress related transcription factors Nrf2, milk fat synthesis-related transcription factors (SREBP1 and PPARG) and milk fat synthases (ACACA and FASN) were measured after BMECs were treated with 50 μM H_2_O_2_ for 1, 3 and 6 h and 1, 10, 50 and 100 μM H_2_O_2_ for 6 h. GAPDH was the housekeeping gene and the data are presented as means ± standard error of mean (SEM). Experiments were repeated three times independently with triplicates in each treatment. * *p* ≤ 0.05, significantly different from CON (0 h).

SREBP1 and PPARG are two important transcription factors that regulate milk fat synthesis. The mRNA abundance of SREBP1 in BMECs was significantly decreased by 8 μg/mL LPS treatment for different times, and the mRNA expression of PPARG was also remarkably decreased by LPS treatment for 8 h. For the key enzymes of fatty acid metabolism, the mRNA abundance of ACACA, FASN and SCD was distinctly decreased between 4 h and 12 h after LPS treatment. According to the above results (Figure 6a), cells were treated with 8 μg/mL LPS for 12 h in subsequent experiments.

To study the effect of oxidative stress on lipid metabolism, the cells were treated with 50 μM H_2_O_2_ for 1 h, 3 h and 6 h and 1, 10, 50 and 100 μM H_2_O_2_ for 6 h (Figure 6b). The mRNA expressions of oxidative stress related transcription factors Nrf2, milk fat synthesis transcription factors SREBP1 and PPARG, and milk fat synthases ACACA and FASN in BMECs treated with 50 μM and 100 μM H_2_O_2_ for 6 h were significantly decreased compared with the control group.

### 3.5. CLA Treatment Promoted Fatty Acid Synthesis

**Figure 7:**
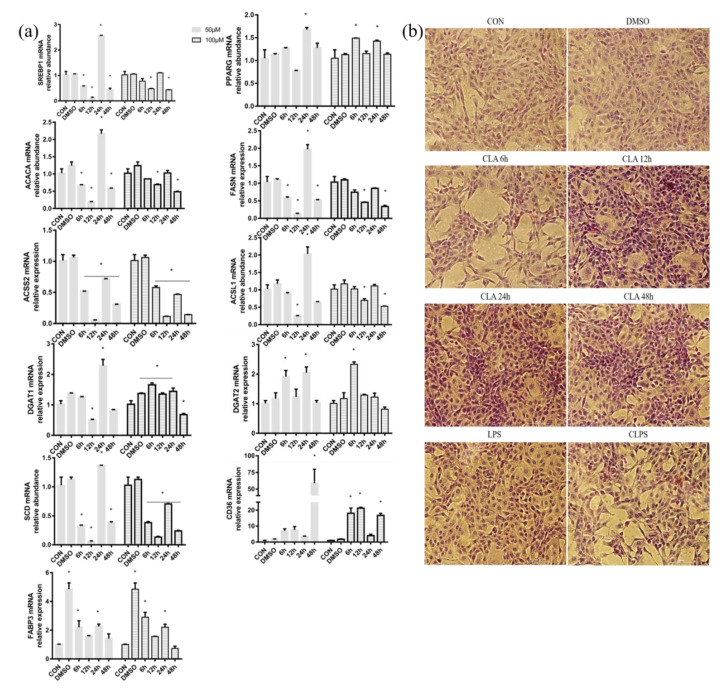
CLA treatment increased the mRNA abundance of milk fat synthesis-related genes and triglyceride synthesis (oil red O staining) in BMECs. (**a**) mRNA expression of transcription factors (SREBP1 and PPARG), de novo synthase (ACACA, FASN and ACSS2), long-chain fatty acid converting enzymes (ACSL1 and SCD), fatty acid transporters (CD36 and FABP3) and triglyceride synthase (DGAT1 and DGAT2) were measured under 50 and 100μM cis-9, trans-11 CLA treatment in BMECs for 6, 12, 24 and 48 h. GAPDH was the housekeeping gene. DMSO was the reagent control group treated with 0.1% dimethyl sulfoxide (DMSO). The data are presented as the means ± SEM; *n* =3 per group. * represented significant differences compared with CON. (**b**) 50 μM CLA was used to treat BMECs for 6 h, 12 h, 24 h and 48 h. The lipid droplets in BMECs were stained orange-red, and the nuclei were counterstained blue with hematoxylin by an oil red O staining kit.

Compared with the control group, 50 and 100 μM CLA treatment for different times in BMECs was found to have different effects on milk fat synthase. Furthermore, 50 μM CLA treatment for 6 h, 12 h and 48 h could significantly reduce the mRNA abundance of transcription factor SREBP1 and milk fat synthase ACACA, FASN, ACSS2 and SCD. However, the mRNA abundance of SREBP1, PPARG, ACACA, FASN, ACSL1, DGAT1, DGAT2, SCD and FABP3 was significantly increased after the 24-h treatment, and the mRNA abundance of CD36 was significantly increased after the 48-h treatment. For the 100 μM CLA treatment, PPARG (for 6 h and 12 h), DGAT1 (for 6–24 h), DGAT2 (for 6 h), CD36 (for 6 h, 12 h and 48 h) and FABP3 (for 6 h and 24 h) were significantly increased for different treatment time, respectively (Figure 7a). Combined with the above results, 24 h 50 μM CLA treatment was used in subsequent experiments.

The lipid droplets in BMECs were stained orange-red, and the nuclei were counterstained blue with hematoxylin by an oil red O staining kit. There were a few orange-red lipid droplets in the cells without any treatment and cells treated with DMSO. With the extension of 50μM CLA treatment time, the number of lipid droplets in BMECs gradually increased, and a large number of lipid droplets were observed in the cells treated with CLA for 12 h and 24 h. However, LPS stimulation could significantly reduce the production of lipid droplets in BMECs (Figure 7b). In conclusion, CLA pretreatment for 24 h can increase the number of lipid droplets in BMECs challenged by LPS for 12 h.

### 3.6. CLA Reversed the Depression of Fatty Acid Caused by LPS

**Figure 8:**
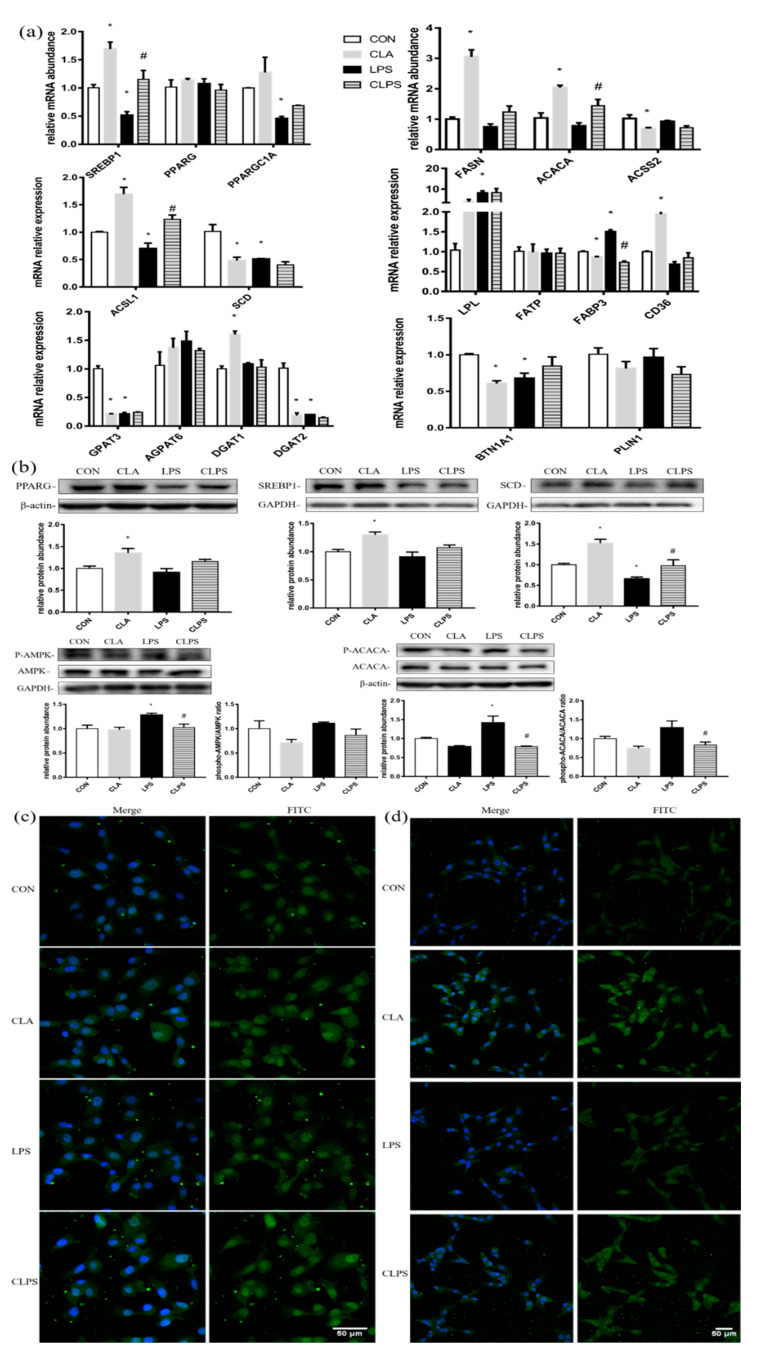
CLA pretreatment relieved the depression of fatty acid synthesis induced by LPS challenge in BMECs. (**a**)The mRNA abundance of transcription factors (SREBP1, PPARG and PPARGC1A), de novo synthase (ACACA, FASN and ACSS2), long-chain fatty acid converting enzymes (ACSL1 and SCD), fatty acid transporters (CD36, LPL, FATP and FABP3), triglyceride synthase (GPAT3, AGPAT6, DGAT1 and DGAT2) and lipid droplet-releasing enzyme (BTN1A1 and PLIN1). (**b**) Immunoblotting of fatty acid synthesis-related transcription factors (SREBP1 and PPARG), enzymes (SCD and ACACA) and AMPK by Western blotting. (**c**) Immunofluorescence of PPARG and (**d**) SREBP1 stained with FITC fluorescence (green) were visualized by laser confocal microscopy. Nuclei were stained with DAPI (blue). GAPDH was the housekeeping gene and GAPDH or β-actin was the reference proteins. The results are presented as means ± SEM. * *p* ≤ 0.05, significantly different from CON; # *p* ≤ 0.05, significantly different from LPS. Experiments were repeated three times independently with triplicates in each treatment.

The 8 μg/mL LPS stimulation in BMECs significantly reduced the mRNA expression of SREBP1, PPARG coactivator 1 alpha (PPARGC1A), ACSL1, SCD, GPAT3, DGAT2 and BTN1A1. CLA treatment alone significantly increased the expression of SREBP1, FASN, ACACA, ACSL1, CD36 and DGAT1. Compared with the LPS group, CLA pretreatment significantly increased the expression of SREBP1, ACACA and ACSL1 inhibited by LPS (Figure 8a).

Compared with the CON group, CLA treatment alone significantly increased the protein expression of SREBP1, PPARG and SCD, while LPS treatment remarkably reduced the protein abundance of SCD, and CLA pretreatment could alleviate the effect of LPS on SCD. LPS stimulation significantly increased the protein abundance of phosphorylated AMPK and the ratio of phosphorylated AMP-activated protein kinase (AMPK) to the total AMPK, while CLA pretreatment significantly decreased the phosphorylated AMPK protein expression. LPS treatment also significantly upregulated the protein abundance of phosphorylated ACACA and the ratio of phosphorylated ACACA to the total ACACA, while CLA pretreatment can significantly reversed the changes caused by LPS challenge (Figure 8b).

CLA treatment alone enhanced the fluorescence intensity of SREBP1 combined with aggregation towards the nucleus. LPS stimulation significantly reduced the fluorescence intensity of SREBP1, and the imagined aggregation towards the nucleus disappeared. In the CLPS group, the fluorescence intensity of SREBP1 was stronger than that of the LPS group (Figure 8d). Compared with the distribution of SREBP1, PPARG was always located in the nucleus and was not affected by various treatments, while the overall fluorescence intensity was affected by various treatments. CLA treatment alone enhanced the fluorescence intensity of PPARG, while LPS treatment reduced the fluorescence intensity of PPARG. The fluorescence intensity of PPARG in the CLPS group was stronger than that in the LPS group (Figure 8c).

## 4. Discussion

Inflammatory response and oxidative stress are two common consequences of LPS challenge. LPS induced the inflammatory response in BMECs, which has been reported in our previous work [32]. After 1 µg/mL LPS stimulation for 1 and 6 h, the ROS content was increased and GSH content was decreased significantly in the bovine mammary alveolar cell line (MAC-T). The expression levels of HMOX1, NQO-1 and TXNRD in MAC-cells were also significantly decreased after 6 h of LPS treatment [37]. In this experiment, LPS challenge increased the concentration of ROS and TBARS, which reflects the degree of lipid peroxidation to some extent, and decreased the concentration of GSH, which was the antioxidant bioactive substance. LPS also depressed the transcription factor Nrf2-regulated anti-oxidative system. In conclusion, LPS stimulation induced the occurrence of oxidative stress in BMECs.

The supplementation of CLA isomers ahead of LPS intramammary challenge to dairy cows not only increased the concentration of glucose in plasma and the usage of beta- hydroxybutyrate as an energy source, but also elevated the temperature change and shortened the time of temperature change, meaning that CLA had an immunomodulatory effect [19]. The diet supplementation of CLA had a positive effect on inflammation and oxidative stress in atherosclerotic patients [38]. However, the antioxidative ability of mixed CLA containing equal amounts of trans-10, cis-12 CLA and cis-9, trans-11 CLA was marginal in lactating dairy cows [20]. Another study reported that cis-9, trans-11 CLA increased the intracellular GSH and NAPDH concentrations and γ-glutamyl-cysteine ligase activity, and decreased the intracellular TBARS concentration, showing a better antioxidant ability than other essential fatty acids [39]. In addition to being an important component of the antioxidant system, the activation of the Nrf2 signaling pathway can also suppress pro-inflammatory cytokines and the activation of NF-κB through the interaction with inflammatory mediators and the NF-κB in various inflammatory diseases [40,41]. Therefore, pretreatment with CLA reduced the imbalance of redox homeostasis caused by LPS through the Nrf2 signaling pathway in BMECs.

Autophagy might be a part of innate immunity, which defends against the invasion of pathogenic bacteria [42]. Impaired autophagy contributes to the susceptibility to mastitis of dairy cows [43]. In this experiment, the autophagy level as a part of the defense system was increased by LPS stimulation, and the inhibition of autophagy exacerbated pro-inflammatory cytokines secretion. Rapamycin, which is an inhibitor of TOR (target of rapamycin), can induce autophagy occurrence in yeast [44] and mammalian cells [45], as mTOR can inhibit autophagy [46]. Rapamycin is also applied as an immunosuppressive agent in clinical [47]. In this experiment, due to the contradictory result on the effect of rapamycin on inflammation, further inflammatory indicators and protein level testing is needed. Autophagy can also protect retinal pigmented epithelial cells against damage caused by oxidative stress [48]. Excessive accumulation of ROS could stimulate the occurrence of autophagy partially through ROS-Nrf2-p62-autophagy signal pathway, meanwhile autophagy in turn could alleviate the oxidative stress in some pathological conditions [49]. In LPS and d-galactosamine induced-acute liver injury model, Nrf2 and autophagy have a complementary relationship to play a role in hepatoprotective activity [50]. Nrf2 regulates the process of autophagy under the condition of oxidative stress combined with AMPK and mTOR [51]. Several studies also report that the prolonged activation of Nrf2 is due to p62 accumulation or deficient autophagy through the direct combination of p62 and Keap1, which inhibits the ubiquitylation degradation of Nrf2 [52,53]. On the basis of the synergistic interaction between Nrf2 and autophagy, both of them are responsible for maintaining the cellular homeostasis of BMECs. The autophagy level was increased by LPS challenge, and cis-9, trans-11 CLA pretreatment could further help BMECs to restore cellular homeostasis through Nrf2 signal pathway.

The intracellular concentration of triglyceride (TG) and non-esterified fatty acids (NEFAs) in MAC-T cells was decreased by LPS stimulation through the inhibition of fatty acid-related genes, ACACA, FASN and SCD [54]. The mRNA and protein abundance of nuclear factor PPARG were downregulated, and its nuclear translocation was restrained by LPS with the depression of its target genes expression [55]. LPS also suppressed the expression and nuclear translocation of another important nuclear factor, SREBP1 [16]. Intramammary infusion with *S. uberis* to the mammary gland caused a potent activation of inflammation and led to a marked prohibition of lipid synthesis, revealing the inverse relationship between the inflammation and synthesis of milk fat [56]. A low dose of LPS (0.1 ng/mL and 1 ng/mL) increased the number of hypermethylated genes belonging to lipid metabolism, whereas high dose of LPS (100 ng/mL) increased the number of hypomethylated genes involved in the immune system, as we know that the methylation degree of gene has a negative relationship with the gene expression. [57]. The occurrence of inflammation and oxidative stress indicated that BMECs were in a stressed state which usually result in cell malfunction or homeostatic imbalance [58]. In this experiment, LPS and H_2_O_2_, a member of ROS, could both depress the fatty acid synthesis-related transcription factors and enzymes, meaning that milk fat depression in dairy cows can be attributed to inflammation and oxidative stress caused by LPS.

Most of research studying the effect of CLA on fatty acid synthesis concerns trans-10, cis-12 CLA, which has been reported to reduce the mRNA and protein expression of fatty acid metabolism-related enzymes in MAC-T cells [59], lactating dairy cows [26] and lactating ewes [60]. CLA production, which contains equal amounts of ruminal- resistant biohydrogenation CLA isomers, tended to increased milk fat yield and milk yield [61]. When luciferase reporter constructs containing individual nuclear factor SREBP-1, PPARG and liver X receptor were transfected into MAC-T, lower concentrations (25 uM) of cis 9, trans 11-CLA inhibited the activation of sterol response element (SRE) and liver X receptor response element (LXRE), but higher concentrations (50–100 uM) of cis 9, trans 11-CLA induced the activation of SRE, peroxisome proliferator response element (PPRE) and LXRE [62]. Based on the ambiguous and limited research on the effect of cis-9, trans-11 CLA on fatty acid metabolism, in this experiment, cis-9, trans-11 CLA could promote milk fat synthesis-related gene expression and lipid droplet formation in BMECs, which is closely related to the concentration and treatment time of CLA. Transcription factor Nrf2 also participates in the regulation of lipid metabolism. The deletion of Nrf2 in mice contributed to the reduction in the liver weight and the triacylglycerol content in the liver along with the downregulation of lipid synthesis and uptake genes [63]. However, Nrf2 can relieve lipid accumulation especially in young animals suffering from nonalcoholic fatty liver disease [64]. Another study reported that Nrf2 promoted lipid accumulation in the white adipose tissue of mice through the upregulating of SREBP1 [65]. Therefore, we speculate that the mechanisms mediating the upregulation of milk fat synthesis-related genes and lipid droplets formation by cis-9, trans-11 CLA is through the upregulation of Nrf2 and the restoration of cellular homeostasis in BMECs.

Dimethyl sulfoxide (DMSO), a low molecular weight chemical chaperone [66], and is commonly used as a solvent for a chemical reagent. In this experiment, DMSO as a reagent control could upregulate one of lipid transporters, FABP3, at the transcript level, with no effect on other genes. One study reported that 0.01% DMSO can decrease triglyceride accumulation by increasing the autophagy level in hepatocytes [67], indicating that DMSO has a relationship with lipid metabolism. However, the study of the effect of DMSO on FABP3 is limited and DMSO was identified to have no effect on lipid metabolism in this study. Further investigation is needed to confirm whether DMSO can regulate lipid metabolism.

## 5. Conclusions

In conclusion, LPS challenge induced oxidative stress and increased the autophagy level, which indicated the disruption of cell homeostasis, thus resulting in the depression of milk fat synthesis. However, cis-9, trans-11 CLA alleviated oxidative stress and autophagy, and promoted milk fat synthesis through the Nrf2 signaling pathway in bovine mammary epithelial cells. We propose a natural therapeutic strategy for mastitis caused by LPS as an alternative to antibiotics.

## Data Availability

All data is comprised in this manuscript.

## Supplementary Materials

The following are available online at https://www.mdpi.com/article/10.3390/antiox11010055/s1, Figure S1. Exploration of treatment time of LPS and CLA on mRNA abundance of anti-oxidative genes in BMECs; Table S1. Determination of CAT, GSH, TBARS and SOD; Table S2. Primers used in quantitative real-time PCR analysis; Table S3. Information of antibodies used in western-blot analysis.

## Abbreviations

ACACA	Acetyl-CoA carboxylase alpha
ACSL1	Acyl-CoA synthetase long-chain family member 1
ACSS2	Acyl-CoA synthetase short chain family member 2
AGPAT6	1-acylglycerol-3-phosphate O-acyltransferase 6
AMPK	AMP-activated protein kinase
ATG5	autophagy related 5
BMECs	Bovine mammary gland epithelial cells
BTN1A1	Butyrophilin subfamily 1 member A1
CAT	Catalase
CLA	Conjugated linoleic acid
CUL3	Cullin 3
DGAT1	Diacylglycerol O-acyltransferase 1
DGAT2	Diacylglycerol O-acyltransferase 2
FABP3	Fatty acid binding protein 3
FASN	Fatty acid synthase
FATP-1	SLC27A1, solute carrier family 27 member 1;
GAPDH	Glyceraldehyde-3-phosphate dehydrogenase
GCLC	Glutamate-cysteine ligase catalytic subunit
GCLM	Glutamate-cysteine ligase modifier subunit
GPAT3	Glycerol-3-phosphate acyltransferase 3
GPX1	Glutathione peroxidase 1;
GSH	Glutathione
GSR	Glutathione-disulfide reductase
GSS	Glutathione synthetase
GSTM1	Glutathione S-transferase mu 1
GSTP	Glutathione S-transferase pi 1
HMOX1	Heme oxygenase 1
IL10	Interleukin 10
IL6	Interleukin 6
IL8	Interleukin 8
KEAP1	Kelch like ECH associated protein 1
LC3	MAP1LC3B, microtubule associated protein 1 light chain 3 beta;
LPIN1	Lipin 1
LPL	Lipoprotein lipase
LPS	Lipopolysaccharide
ME1	Malic enzyme 1
Nrf2	Nuclear factor erythroid 2 like 2
NF-κB	Nuclear factor κB
NQO1	NAD(P)H quinone dehydrogenase 1
p62	SQSTM1, sequestosome 1
PLIN1	perilipin 1
PPARG	Peroxisome proliferator activated receptor gamma
PPARGC1A	PPARG coactivator 1 alpha
ROS	Reactive oxygen species
SCD	Stearoyl-CoA desaturase
SOD	Superoxide dismutase
SREBP1	Sterol regulatory element binding protein
T-AOC	Total antioxidant capacity
TBARS	Thiobarbituric acid-reactive substances
TXN	Thioredoxin
TXNRD	Thioredoxin reductase

## References

[1] Deb R., Kumar A., Chakraborty S., Verma A., Tiwari R., Dhama K., Singh U., Kumar S. (2013). Trends in diagnosis and control of bovine mastitis: A review. Pak. J. Biol. Sci..

[2] Bradley A. (2002). Bovine mastitis: An evolving disease. Vet. J..

[3] Lippolis J.D., Holman D.B., Brunelle B.W., Thacker T.C., Bearson B.L., Reinhardt T.A., Sacco R.E., Casey T.A. (2018). Genomic and Transcriptomic Analysis of Escherichia coli Strains Associated with Persistent and Transient Bovine Mastitis and the Role of Colanic Acid. Infect. Immun..

[4] Hogan J., Smith K.L. (2003). Coliform mastitis. Vet. Res..

[5] Kan X., Liu B., Guo W., Wei L., Lin Y., Guo Y., Gong Q., Li Y., Xu D., Cao Y. (2019). Myricetin relieves LPS-induced mastitis by inhibiting inflammatory response and repairing the blood-milk barrier. J. Cell. Physiol..

[6] Raetz C.R., Whitfield C. (2002). Lipopolysaccharide endotoxins. Annu. Rev. Biochem..

[7] Lu Y.C., Yeh W.C., Ohashi P.S. (2008). LPS/TLR4 signal transduction pathway. Cytokine.

[8] Castillo C., Hernandez J., Bravo A., Lopez-Alonso M., Pereira V., Benedito J.L. (2005). Oxidative status during late pregnancy and early lactation in dairy cows. Vet. J..

[9] Mavangira V., Sordillo L.M. (2018). Role of lipid mediators in the regulation of oxidative stress and inflammatory responses in dairy cattle. Res. Vet. Sci..

[10] Smith K.L., Hogan J.S., Weiss W.P. (1997). Dietary vitamin E and selenium affect mastitis and milk quality. J. Anim. Sci..

[11] Rahman I., Biswas S.K., Jimenez L.A., Torres M., Forman H.J. (2005). Glutathione, stress responses, and redox signaling in lung inflammation. Antioxid. Redox Signal..

[12] Kensler T.W., Wakabayashi N., Biswal S. (2007). Cell survival responses to environmental stresses via the Keap1-Nrf2-ARE pathway. Annu. Rev. Pharmacol. Toxicol..

[13] Schaaf M.B., Houbaert D., Meçe O., Agostinis P. (2019). Autophagy in endothelial cells and tumor angiogenesis. Cell Death Differ..

[14] Wang Z., Li Z., Feng D., Zu G., Li Y., Zhao Y., Wang G., Ning S., Zhu J., Zhang F. (2018). Autophagy Induction Ameliorates Inflammatory Responses in Intestinal Ischemia-Reperfusion Through Inhibiting NLRP3 Inflammasome Activation. Shock.

[15] Li X., Li G., Du X., Sun X., Peng Z., Zhao C., Xu Q., Abdelatty A.M., Mohamed F.F., Wang Z. (2020). Increased autophagy mediates the adaptive mechanism of the mammary gland in dairy cows with hyperketonemia. J. Dairy Sci..

[16] Peng X., Wang Y., Li H., Fan J., Shen J., Yu X., Zhou Y., Mao H. (2019). ATG5-mediated autophagy suppresses NF-kappaB signaling to limit epithelial inflammatory response to kidney injury. Cell Death Dis..

[17] Kim J.H., Kim Y., Kim Y.J., Park Y. (2016). Conjugated Linoleic Acid: Potential Health Benefits as a Functional Food Ingredient. Annu. Rev. Food Sci. Technol..

[18] O’Shea M., Bassaganya-Riera J., Mohede I.C. (2004). Immunomodulatory properties of conjugated linoleic acid. Am. J. Clin. Nutr..

[19] Gross J.J., Grossen-Rösti L., Héritier R., Tröscher A., Bruckmaier R.M. (2018). Inflammatory and metabolic responses to an intramammary lipopolysaccharide challenge in early lactating cows supplemented with conjugated linoleic acid. J. Anim. Physiol. Anim. Nutr..

[20] Hanschke N., Kankofer M., Ruda L., Höltershinken M., Meyer U., Frank J., Dänicke S., Rehage J. (2016). The effect of conjugated linoleic acid supplements on oxidative and antioxidative status of dairy cows. J. Dairy Sci..

[21] Basiricò L., Morera P., Dipasquale D., Tröscher A., Serra A., Mele M., Bernabucci U. (2015). Conjugated linoleic acid isomers strongly improve the redox status of bovine mammary epithelial cells (BME-UV1). J. Dairy Sci..

[22] Bergamo P., Palmieri G., Cocca E., Ferrandino I., Gogliettino M., Monaco A., Maurano F., Rossi M. (2016). Adaptive response activated by dietary cis9, trans11 conjugated linoleic acid prevents distinct signs of gliadin-induced enteropathy in mice. Eur. J. Nutr..

[23] Bergamo P., Maurano F., D’Arienzo R., David C., Rossi M. (2008). Association between activation of phase 2 enzymes and down-regulation of dendritic cell maturation by c9, t11-conjugated linoleic acid. Immunol. Lett..

[24] Zebeli Q., Ametaj B.N. (2009). Relationships between rumen lipopolysaccharide and mediators of inflammatory response with milk fat production and efficiency in dairy cows. J. Dairy Sci..

[25] Wang J., Zhang X., He X., Yang B., Wang H., Shan X., Li C., Sun D., Wu R. (2018). LPS-induced reduction of triglyceride synthesis and secretion in dairy cow mammary epithelial cells via decreased SREBP1 expression and activity. J. Dairy Res..

[26] Harvatine K.J., Bauman D.E. (2006). SREBP1 and thyroid hormone responsive spot 14 (S14) are involved in the regulation of bovine mammary lipid synthesis during diet-induced milk fat depression and treatment with CLA. J. Nutr..

[27] Baumgard L.H., Matitashvili E., Corl B.A., Dwyer D.A., Bauman D.E. (2002). Trans-10, cis-12 conjugated linoleic acid decreases lipogenic rates and expression of genes involved in milk lipid synthesis in dairy cows. J. Dairy Sci..

[28] Chouinard P.Y., Corneau L., Barbano D.M., Metzger L.E., Bauman D.E. (1999). Conjugated linoleic acids alter milk fatty acid composition and inhibit milk fat secretion in dairy cows. J. Nutr..

[29] Lin X., Loor J.J., Herbein J.H. (2004). Trans10, cis12-18:2 is a more potent inhibitor of de novo fatty acid synthesis and desaturation than cis9,trans11-18:2 in the mammary gland of lactating mice. J. Nutr..

[30] Mollica M.P., Trinchese G., Cavaliere G., De Filippo C., Cocca E., Gaita M., Della-Gatta A., Marano A., Mazzarella G., Bergamo P. (2014). c9,t11-Conjugated linoleic acid ameliorates steatosis by modulating mitochondrial uncoupling and Nrf2 pathway. J. Lipid Res..

[31] Zhao K., Liu H.Y., Zhou M.M., Liu J.X. (2010). Establishment and characterization of a lactating bovine mammary epithelial cell model for the study of milk synthesis. Cell Biol. Int..

[32] Ma N., Chang G., Huang J., Wang Y., Gao Q., Cheng X., Liu J., Shen X. (2019). cis-9, trans-11-Conjugated Linoleic Acid Exerts an Anti-inflammatory Effect in Bovine Mammary Epithelial Cells after Escherichia coli Stimulation through NF-κB Signaling Pathway. J. Agric. Food Chem..

[33] Gao Q., Wang Y., Ma N., Dai H., Roy A.C., Chang G., Shi X., Shen X. (2020). Sodium valproate attenuates the iE-DAP induced inflammatory response by inhibiting the NOD1-NF-κB pathway and histone modifications in bovine mammary epithelial cells. Int. Immunopharmacol..

[34] Cheng X., Aabdin Z.U., Wang Y., Ma N., Dai H., Shi X., Shen X. (2021). Glutamine pretreatment protects bovine mammary epithelial cells from inflammation and oxidative stress induced by γ-d-glutamyl-meso-diaminopimelic acid (iE-DAP). J. Dairy Sci..

[35] Janero D.R. (1990). Malondialdehyde and thiobarbituric acid-reactivity as diagnostic indices of lipid peroxidation and peroxidative tissue injury. Free Radic. Biol. Med..

[36] Livak K.J., Schmittgen T.D. (2001). Analysis of relative gene expression data using real-time quantitative PCR and the 2(-Delta Delta C (T)) Method. Methods.

[37] Fusco R., Cordaro M., Siracusa R., Peritore A.F., D’Amico R., Licata P., Crupi R., Gugliandolo E. (2020). Effects of Hydroxytyrosol against Lipopolysaccharide-Induced Inflammation and Oxidative Stress in Bovine Mammary Epithelial Cells: A Natural Therapeutic Tool for Bovine Mastitis. Antioxidants.

[38] Hassan Eftekhari M., Aliasghari F., Babaei-Beigi M.A., Hasanzadeh J. (2013). Effect of conjugated linoleic acid and omega-3 fatty acid supplementation on inflammatory and oxidative stress markers in atherosclerotic patients. ARYA Atheroscler..

[39] Basiricò L., Morera P., Dipasquale D., Tröscher A., Bernabucci U. (2017). Comparison between conjugated linoleic acid and essential fatty acids in preventing oxidative stress in bovine mammary epithelial cells. J. Dairy Sci..

[40] Ahmed S.M., Luo L., Namani A., Wang X.J., Tang X. (2017). Nrf2 signaling pathway: Pivotal roles in inflammation. Biochimica et biophysica acta. Mol. Basis Dis..

[41] Saha S., Buttari B., Panieri E., Profumo E., Saso L. (2020). An Overview of Nrf2 Signaling Pathway and Its Role in Inflammation. Molecules.

[42] Amano A., Nakagawa I., Yoshimori T. (2006). Autophagy in innate immunity against intracellular bacteria. J. Biochem..

[43] Sugimoto M., Sugimoto Y. (2012). Variant in the 5′ untranslated region of insulin-like growth factor 1 receptor is associated with susceptibility to mastitis in cattle. G3.

[44] Noda T., Ohsumi Y. (1998). Tor, a phosphatidylinositol kinase homologue, controls autophagy in yeast. J. Biol. Chem..

[45] Sato M., Seki T., Konno A., Hirai H., Kurauchi Y., Hisatsune A., Katsuki H. (2019). Rapamycin activates mammalian microautophagy. J. Pharmacol. Sci..

[46] Nazio F., Strappazzon F., Antonioli M., Bielli P., Cianfanelli V., Bordi M., Gretzmeier C., Dengjel J., Piacentini M., Fimia G.M. (2013). mTOR inhibits autophagy by controlling ULK1 ubiquitylation, self-association and function through AMBRA1 and TRAF6. Nat. Cell Biol..

[47] Soliman G.A. (2013). The role of mechanistic target of rapamycin (mTOR) complexes signaling in the immune responses. Nutrients.

[48] Mitter S.K., Song C., Qi X., Mao H., Rao H., Akin D., Lewin A., Grant M., Dunn W., Ding J. (2014). Dysregulated autophagy in the RPE is associated with increased susceptibility to oxidative stress and AMD. Autophagy.

[49] Li L., Tan J., Miao Y., Lei P., Zhang Q. (2015). ROS and Autophagy: Interactions and Molecular Regulatory Mechanisms. Cell. Mol. Neurobiol..

[50] Lv H., Yang H., Wang Z., Feng H., Deng X., Cheng G., Ci X. (2019). Nrf2 signaling and autophagy are complementary in protecting lipopolysaccharide/d-galactosamine-induced acute liver injury by licochalcone A. Cell Death Dis..

[51] Kapuy O., Papp D., Vellai T., Bánhegyi G., Korcsmáros T. (2018). Systems-Level Feedbacks of NRF2 Controlling Autophagy upon Oxidative Stress Response. Antioxidants.

[52] Jiang T., Harder B., Rojo de la Vega M., Wong P.K., Chapman E., Zhang D.D. (2015). p62 links autophagy and Nrf2 signaling. Free Radic. Biol. Med..

[53] Komatsu M., Kurokawa H., Waguri S., Taguchi K., Kobayashi A., Ichimura Y., Sou Y.-S., Ueno I., Sakamoto A., Tong K.I. (2010). The selective autophagy substrate p62 activates the stress responsive transcription factor Nrf2 through inactivation of Keap1. Nat. Cell Biol..

[54] Wu Y., Sun Y., Zhang Z., Chen J., Dong G. (2020). Effects of Peptidoglycan, Lipoteichoic Acid and Lipopolysaccharide on Inflammation, Proliferation and Milk Fat Synthesis in Bovine Mammary Epithelial Cells. Toxins.

[55] He X.J., Lian S., Zhang X., Hao D.D., Shan X.F., Wang D., Sun D.B., Wu R., Wang J.F. (2019). Contribution of PPAR gamma in Modulation of LPS-Induced Reduction of Milk Lipid Synthesis in Bovine Mammary Epithelial Cells. Int. J. Agric. Biol..

[56] Moyes K.M., Drackley J.K., Morin D.E., Bionaz M., Rodriguez-Zas S.L., Everts R.E., Lewin H.A., Loor J.J. (2009). Gene network and pathway analysis of bovine mammary tissue challenged with Streptococcus uberis reveals induction of cell proliferation and inhibition of PPARgamma signaling as potential mechanism for the negative relationships between immune response and lipid metabolism. BMC Genom..

[57] Chen J., Wu Y., Sun Y., Dong X., Wang Z., Zhang Z., Xiao Y., Dong G. (2019). Bacterial Lipopolysaccharide Induced Alterations of Genome-Wide DNA Methylation and Promoter Methylation of Lactation-Related Genes in Bovine Mammary Epithelial Cells. Toxins.

[58] Medzhitov R. (2008). Origin and physiological roles of inflammation. Nature.

[59] Peterson D.G., Matitashvili E.A., Bauman D.E. (2004). The inhibitory effect of trans-10, cis-12 CLA on lipid synthesis in bovine mammary epithelial cells involves reduced proteolytic activation of the transcription factor SREBP-1. J. Nutr..

[60] Hussein M., Harvatine K.H., Weerasinghe W.M., Sinclair L.A., Bauman D.E. (2013). Conjugated linoleic acid-induced milk fat depression in lactating ewes is accompanied by reduced expression of mammary genes involved in lipid synthesis. J. Dairy Sci..

[61] Oliveira R.C., Pralle R.S., de Resende L.C., Nova C., Caprarulo V., Jendza J.A., Troescher A., White H.M. (2018). Prepartum supplementation of conjugated linoleic acids (CLA) increased milk energy output and decreased serum fatty acids and β-hydroxybutyrate in early lactation dairy cows. PLoS ONE.

[62] Ma L., Lengi A.J., McGilliard M.L., Bauman D.E., Corl B.A. (2014). Short communication: Effect of trans-10,cis-12 conjugated linoleic acid on activation of lipogenic transcription factors in bovine mammary epithelial cells. J. Dairy Sci..

[63] Huang J., Tabbi-Anneni I., Gunda V., Wang L. (2010). Transcription factor Nrf2 regulates SHP and lipogenic gene expression in hepatic lipid metabolism. Am. J. Physiol. Gastrointest. Liver Physiol..

[64] Chambel S.S., Santos-Gonçalves A., Duarte T.L. (2015). The Dual Role of Nrf2 in Nonalcoholic Fatty Liver Disease: Regulation of Antioxidant Defenses and Hepatic Lipid Metabolism. BioMed Res. Int..

[65] Sun X., Li X., Jia H., Wang H., Shui G., Qin Y., Shu X., Wang Y., Dong J., Liu G. (2020). Nuclear Factor E2-Related Factor 2 Mediates Oxidative Stress-Induced Lipid Accumulation in Adipocytes by Increasing Adipogenesis and Decreasing Lipolysis. Antioxid. Redox Signal..

[66] Morello J.P., Petäjä-Repo U.E., Bichet D.G., Bouvier M. (2000). Pharmacological chaperones: A new twist on receptor folding. Trends Pharmacol. Sci..

[67] Song Y.M., Song S.O., Jung Y.K., Kang E.S., Cha B.S., Lee H.C., Lee B.W. (2012). Dimethyl sulfoxide reduces hepatocellular lipid accumulation through autophagy induction. Autophagy.

