# Supplementary material for: Cis-9, Trans-11 CLA Alleviates Lipopolysaccharide-Induced Depression of Fatty Acid Synthesis by Inhibiting Oxidative Stress and Autophagy in Bovine Mammary Epithelial Cells
Source: Antioxidants (Basel). 2021 Dec 27;11(1):55. doi: 10.3390/antiox11010055 (PMC8773093; doi:10.3390/antiox11010055)
Supplement: Supplementary file 1 [file antioxidants-11-00055-s001.zip › antioxidants-1470531-supplementary.pdf]

Supplementary Materials

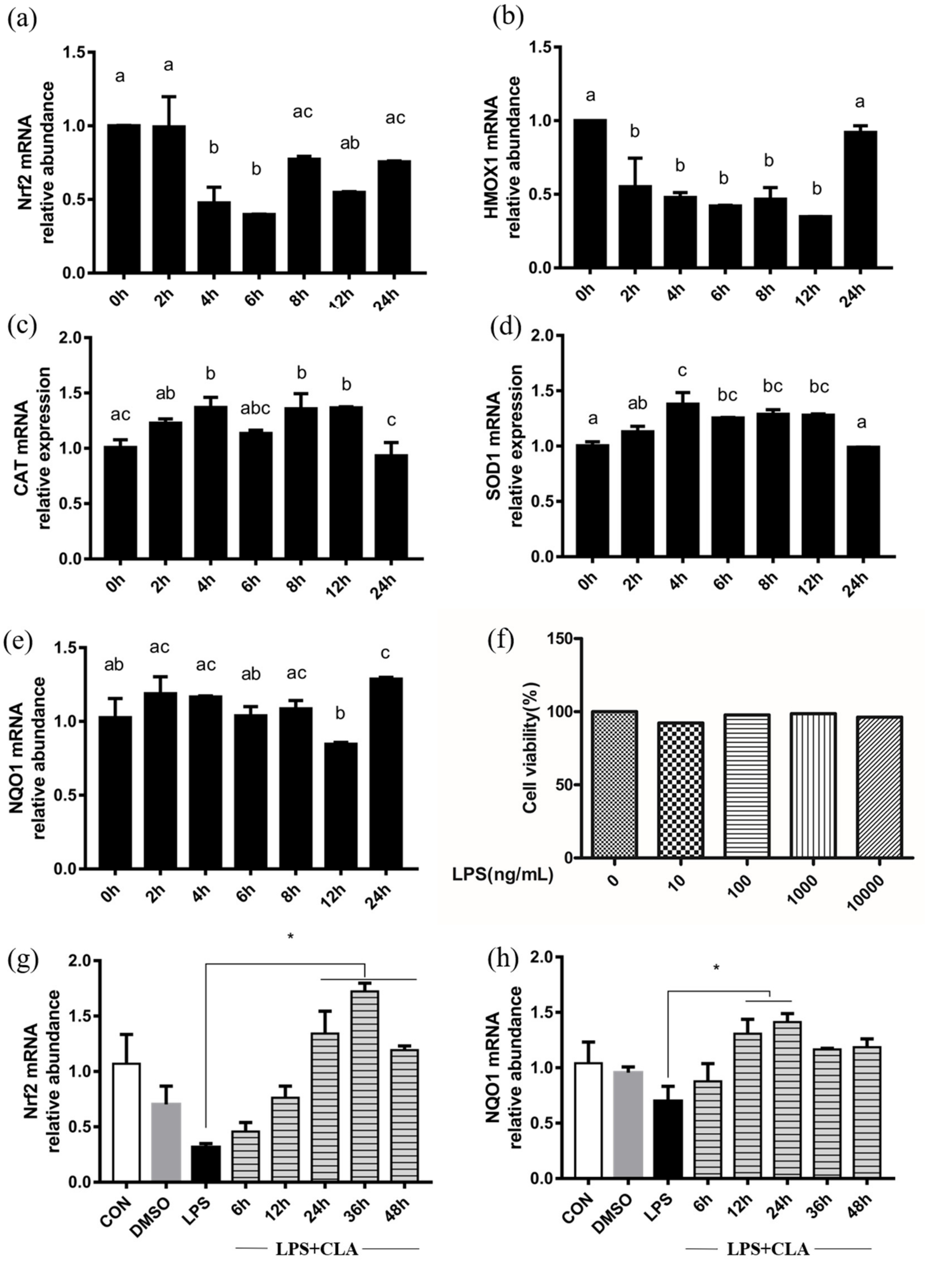

Figure S1. | Exploration of treatment time of LPS and CLA on mRNA abundance of anti-oxidative genes in BMECs. (**a**–**e**) BMECs were treated with 8 μg/mL LPS for 0, 2, 4, 6, 8, 12 and 24 h. (**f**) BMECs were treated with 1, 10, 100, 1000, 10000 ng/mL LPS for 48h to measure cell viability. Cell viability was detected by cell counting kit-8 (CCK-8). (**g**,**h**) BMECs were pretreated with CLA for 6, 12, 24, 36 and 48 h, then followed by treatment with 8 μg/mL LPS for 12h (LPS + CLA group). The gene expression was normalized to the gene expression of GAPDH. The data are presented as the means ± SEM. Different letters represented significant differences between groups ($p < 0.05$). * $p < 0.05$, represented significant differences between LPS group and LPS + CLA group.

Table S1. Determination of CAT, GSH, TBARS and SOD.

Indicators	Description about methods
Catalase (CAT)	The kit uses the ammonium molybdate method, that is, the reaction of CAT decomposing H_2O_2 can be quickly terminated by ammonium molybdate, and the remaining H_2O_2 reacts with ammonium molybdate to form a pale-yellow complex, whose OD value can be measured at 405nm to calculate CAT activity, as described by Goth [68]. Before determination, it is necessary to dilute 10% tissue or cell homogenate with physiological saline or PBS to different concentrations for pre-experiment, the proportional concentration of homogenate was selected for formal experiment. One unit of CAT activity was defined as the amount of enzyme decomposing 1 μmol of H_2O_2 in 1 s. The final result was normalized by protein concentration (mgprot/mL) and the unit of the final result was U/mgprot.
Superoxide Dismutase (SOD)	This detection adopts WST reaction system that contains xanthine; xanthine oxidase produces the superoxide anion free radical ($O_2{\cdot}^-$). WST can react with the superoxide anion in the tissue to produce water-soluble dye WST formazan, and this process can be inhibited by SOD, as described by Ukeda et al. [69]. Before using this kit to test samples, individual samples should be selected and diluted to different concentrations for pre-experiment. A dilution ratio of 40%-60% SOD inhibition rate should be selected for formal test. After each sample was tested according to the kit instruction, the OD value was read at 450nm wavelength. In this reaction system, when the SOD inhibition rate reaches 50%, the amount of enzyme corresponding to one SOD activity unit (U). The limit of detection is 0.5 U/mL. The final result was normalized by protein concentration (mg prot/mL) and the unit of the final result was U/mg prot.
Reduced glutathione (GSH)	Reduced glutathione, a tripeptide of glutamic acid, cysteine, and glycine, is the most important non-enzymatic antioxidant in the body. Cysteine contains a sulfhydryl group (-SH), which can react with DTNB and break the disulfide bond of DTNB to generate yellow compound, 2-nitro-5-thiobenzoic acid (NTB-), as described by Tietze [70]. The OD value of NTB- is measured at 405nm. GSH content is quantitatively determined by colorimetric method. Standard curves were made with different concentrations of standard GSH (100μmol/L, 50μmol/L, 20μmol/L, 10μmol/L, 5μmol/L, 0μmol/L), and sample concentration was calculated according to the standard curves. The sensitivity of the kit is 1.5μmol/L. The final result was normalized by protein concentration (mgprot/mL) and the unit of the final result was μmol/gprot.
Thiobarbituric acid-reactive substances (TBARS)	1. Lipid peroxidation products (thiobarbituric acid-reactive substances) can be combined with thiobarbituric acid (TBA) to form a red product with a maximum absorption peak at 532nm, as described by Pryor [71]. The variable coefficient (CV)= 1.5%. Lipid peroxidation was expressed as nanomoles of MDA per milligram of protein. Since this TBA test has many limitations to reflect the real state of lipid peroxidation, it need combine with other markers to indicate the redox state of tissue or cell.

Table S2. Primers used in quantitative real-time PCR analysis.

Target Gene [1]		Primer sequence	Accession #	Product(bp）
ACACA	Forward	AGCTGAATTTTCGCAGCAAT	NM_174224.2	117
	Reverse	GGTTTTCTCCCCAGGAAAAG		
ACSL1	Forward	GTGGGCTCCTTTGAAGAACTGT	BC119914	120
	Reverse	ATAGATGCCTTTGACCTGTTCAAAT		
ACSS2	Forward	GGCGAATGCCTCTACTGCTT	BC134532	100
	Reverse	GGCCAATCTTTTCTCTAATCTGCTT		
AGPAT6	Forward	AAGCAAGTTGCCCATCCTCA	DY208485	101
	Reverse	AAACTGTGGCTCCAATTTCGA		
BTN1A1	Forward	AGGACGGACTGGGCAATTG	M35551	81
	Reverse	GAACCCATTCTCGGGAGTCAT		
CAT	Forward	TCACTCAGGTGCGGACTTTC	NM_001035386.2	163
	Reverse	TGGATGCGGGAGCCATATTC		
CD36	Forward	GTACAGATGCAGCCTCATTTCC	X91503	81
	Reverse	TGGACCTGCAAATATCAGAGGA		
CUL3	Forward	CTTTCCGATGACCATGG	NM_001192806.2	110
	Reverse	GCTCCTCAAAACTAAGACCACTG		
DGAT1	Forward	CCACTGGGACCTGAGGTGTC	NM_174693	101
	Reverse	GCATCACCACACACCAATTCA		
DGAT2	Forward	CATGTACACATTCTGCACCGATT	BT030532.1	100
	Reverse	TGACCTCCTGCCACCTTTCT		
FABP3	Forward	GAACTCGACTCCCAGCTTGAA	DN518905	102
	Reverse	AAGCCTACCACAATCATCGAAG		
FASN	Forward	GCATCGCTGGCTACTCCTAC	CR552737	92
	Reverse	GTGTAGGCCATCACGAAGGT		
FATP-1	Forward	CTGTACGAGGAACTGCAGAAGGT	NM_001033625.2	98
	Reverse	TGAATTTTGAAGGTGCCTGTAGTG		
GCLC	Forward	ATTGGGTGGAGAGTGGAA	NM_001083674	133
	Reverse	ACAGCGGGATGAGAAAGT		
GCLM	Forward	TGGAGCAGCTGTATCAGTGG	NM_001083674	198
	Reverse	GAATGTCAGGGATGCTCTCC		
GPAT3	Forward	CCCCCATGACCAGAGAGGA	NM_001192514.3	103
	Reverse	CCATCCCAAGGAAGCTCAGTC		
GPX1	Forward	CTTCAACCTGTCCTCCCT	NM_174076.3	98
	Reverse	GGTCATTCATCTGGGTGT		
GSR	Forward	CGCTGAGAACCCAGAG	NM _ 001114190.2	100
	Reverse	AAACGGAAAGTGGGAACAGTAAGTA		
GSS	Forward	CGAGTGATCCAATGCAT	NM_001015630.1	90
	Reverse	ATGTCCCACGTGCTTGTTCAT		
GSTM1	Forward	ATCTGCTACAGCCCTGA	NM _ 175825	100
	Reverse	AAGGCCTCTTCCCCAGAAAC		
GSTP	Forward	TTTGCGGACTACAACCTG	NM_177516.1	186
	Reverse	CCCTCACTGTTTCCCATT		
HMOX1	Forward	GAACGCAACAAGGAGAAC	NM_001014912	162
	Reverse	CTGGAGTCGCTGAACATAG		
IL10	Forward	AGCACTACTCTGTTGCCTGG	NM_174088.1	230
	Reverse	TTGGGGTAGACTTTGGGGTCT		
IL6	Forward	GGAGGAAAAGGACGGATGCT	NM_173923.2	227
	Reverse	GGTCAGTGTTTGTGGCTGGA		
IL-8	Forward	CCTCTTGTTCAATATGACTTCCA	NM_173925.2	170
	Reverse	GGCCCACTCTCAATAACTCTC		
KEAP1	Forward	ACAACAGTGTGGAGAG	NM_001101142.1	108
	Reverse	AGAGCAGACGGTTGAGGACAG		
LPL	Forward	GGGTTTTGAGCAAGGGTACA	NM_001075120.1	193
	Reverse	GCCACAATGACCTTTCCAGT		
ME1	Forward	CCAACTGCCCTCATTGGAGT	NM_001144853	166
	Reverse	CGCGACCCTTGGTCAGTTTA		
Nrf2	Forward	AGGACATGGATTTGATT	NM_001011678.2	272
	Reverse	TACCTGGGAGTAGTTGGCA		
NQO1	Forward	CAACAGACCAGCCAATCA	NM_001034535	144

	Reverse	ACCTCCCATCCTTTCCTC		
PLIN1	Forward	GATCGCCTCTGAGCTGAAGG	DV814745	108
	Reverse	AGAGCGGCCCCTAGGATTT		
PPARG	Forward	GAGCCCAAGTTCGAGTT	NM_181024.2	100
	Reverse	GGCGGTCTCCACTGAGAATAAT		
PPARGC1A	Forward	GTACCAGCACGAAAGGCTCAA	NM_177945	120
	Reverse	ATCACACGGCGCTCTTCAA		
SCD	Forward	TTATTCCGTTATGCCCTTGG	NM_173959.4	151
	Reverse	GGTAGTTGTGGAAGCCCTCA		
SOD	Forward	TGTTGGAGACCTGGGCAATG	NM_174615.2	146
	Reverse	CTCTGCCCAAGTCATCTGGTT		
SREBP1	Forward	CACTCGTCTTCCTCTGTCTC	NM_001113302.1	243
	Reverse	GAGTGACTGGTTCTCCATAG		
TXN	Forward	TAGTCGACTTCTCAGCCACG	NM _ 173968	136
	Reverse	CACTCTGCAGCAACATCCTGA		
TXNRD	Forward	GTGTGAATGTGGGTTGCATACC	NM _ 174625	100
	Reverse	TCCTCGACGTTCCACCCATA		
GAPDH	Forward	GGGTCATCATCTCTGCACCT	NM_001034034	176
	Reverse	GGTCATAAGTCCCTCCACGA		

Table S3. Information of antibodies used in western-blot analysis.

Target protein	Cat. #	Dilution	Category	Manufacture
AMPK	AF1627	1:1000	Primary antibody	Beyotime biotechnology
p-AMPK	AA393	1:800	Primary antibody	Beyotime biotechnology
Nrf2	AF0639	1:500	Primary antibody	Affinity
p-Nrf2	A1609	1:200	Primary antibody	Beyotime biotechnology
HMOX-1	AF1333	1:1000	Primary antibody	Beyotime biotechnology
GAPDH	60004-1-Ig	1:5000	Primary antibody	Proteintech
ACACA	AF1867	1:1000	Primary antibody	Beyotime biotechnology
p-ACACA	AA110	1:1000	Primary antibody	Beyotime biotechnology
PPARG	Bs-0530R	1:500	Primary antibody	Bioss antibodies
SREBP1	AF8055	1:1000	Primary antibody	Beyotime biotechnology
SCD	SC-23016	1:200	Primary antibody	Santa Cruz Biotechnology
β-actin	AF0003	1:1000	Primary antibody	Beyotime biotechnology
Anti-rabbit IgG, HRP-linked Antibody	A0208	1:1000	Secondary antibody	Beyotime biotechnology
Anti-mouse IgG, HRP-linked Antibody	A0216	1:1000	Secondary antibody	Beyotime biotechnology
Anti-goat IgG, HRP-linked Antibody	S0010	1:5000	Secondary antibody	Affinity
FITC conjugated goat anti-rabbit antibody	A0562	1:1000	Secondary antibody	Beyotime biotechnology
FITC conjugated goat anti-mouse antibody	A0568	1:1000	Secondary antibody	Beyotime biotechnology

Reference

68. Góth, L. A simple method for determination of serum catalase activity and revision of reference range. Clinica chimica acta; international journal of clinical chemistry **1991**, *196*, 143–151, doi:10.1016/0009-8981(91)90067-m.
69. Ukeda, H.; Kawana, D.; Maeda, S.; Sawamura, M. Spectrophotometric Assay for Superoxide Dismutase Based on the Reduction of Highly Water-soluble Tetrazolium Salts by Xanthine-Xanthine Oxidase. Bioscience, biotechnology, and biochemistry **1999**, *63*, 485–488, doi:10.1271/bbb.63.485.
70. Tietze, F. Enzymic method for quantitative determination of nanogram amounts of total and oxidized glutathione: Applications to mammalian blood and other tissues. Analytical biochemistry **1969**, *27*, 502–522, doi:10.1016/0003-2697(69)90064-5.
71. Pryor, W.A. On the detection of lipid hydroperoxides in biological samples. Free radical biology & medicine **1989**, *7*, 177–178, doi:10.1016/0891-5849(89)90010-5.
